# Supplementary material for: A Water-Dispersible Carboxylated Carbon Nitride Nanoparticles-Based Electrochemical Platform for Direct Reporting of Hydroxyl Radical in Meat
Source: Foods. 2021 Dec 24;11(1):40. doi: 10.3390/foods11010040 (PMC8750351; doi:10.3390/foods11010040)
Supplement: Supplementary file 1 [file foods-11-00040-s001.zip › foods-1490516-supplementary.pdf]

Supporting Information

A Water-Dispersible Carboxylated Carbon Nitride Nanoparticles-Based Electrochemical Platform for Direct Reporting of Hydroxyl Radical in Meat

Tingting Han [1], Yang Huang [1], Chong Sun [1,2,*], Daoying Wang [1,*] and Weimin Xu [1]

[1] Institute of Agricultural Products Processing, Jiangsu Academy of Agricultural Sciences, Nanjing 210014, China; 2020015041@qymail.bhu.edu.cn (T.H.); 192702014@njnu.edu.cn (Y.H.); xuweimin@jaas.ac.cn (W.X.);

[2] Jiangsu Key Laboratory for Food Quality and Safety-State Key Laboratory Cultivation Base, Ministry of Science and Technology, Nanjing 210014, China

[*] Correspondence: sunchong@jaas.ac.cn (C.S.); wangdaoying@jaas.ac.cn (D.W.)

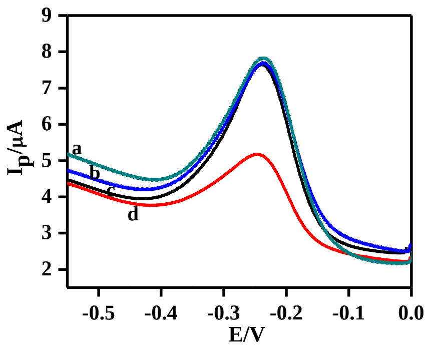

Figure S1. SWV plots of MB in 0.1 M PBS (pH=7.4) obtained from ssDNA/carboxylated-*g*-C_3N_4/chitosan/GCE without 1 mM Fe^{2+} (curve a), with 1 mM Fe^{2+} (curve b), with 6 mM H_2O_2 (curve c), with Fenton reagent (1 mM Fe^{2+} and 6 mM H_2O_2, curve d).

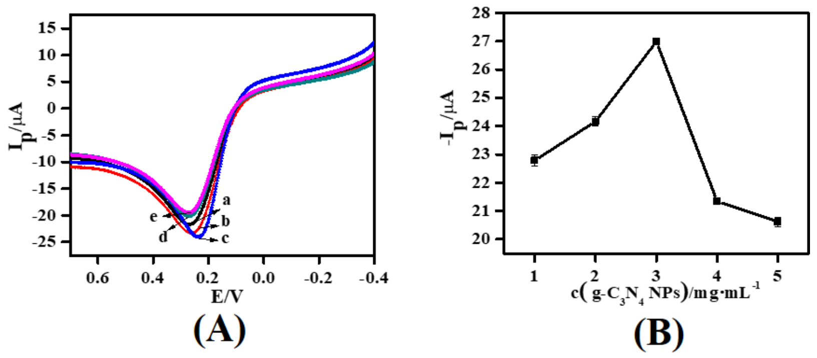

Figure S2. (A) The LSV plots of different concentrations of the carboxylated-*g*-C_3N_4 NPs (a: 1 mg·mL^{-1}, b: 2 mg·mL^{-1}, c: 3 mg·mL^{-1}, d: 4 mg·mL^{-1}, e: 5 mg·mL^{-1}), (B) The effect of the carboxylated-*g*-C_3N_4 NPs concentration in 1 mM $[Fe(CN)_6]^{3-/4-}$ (1:1) solution containing 0.1 M KCl (pH=7.4).

Table S1. Correlation coefficient matrix of sensor evaluation and experimental parameters. Positive coefficients indicated a direct relationship between variables in the matrix. *$p \leqslant 0.05$, **$p \leqslant 0.01$.

	electrochemical signal	The concentrations of ssDNA	The concentrations of MB	The incubation time of carboxylated-*g*-C_3N_4 NPs and amino-terminated ssDNA	The binding time of ssDNA and MB
Electrochemical signal	1				
The concentrations of ssDNA	0.890*	1			
The concentrations of MB	0.864*	0.889*	1		
The incubation time of carboxylated-*g*-C_3N_4 NPs and amino-terminated ssDNA	0.829*	0.919*	0.986**	1	
The binding time of ssDNA and MB	0.935*	0.998**	0.907*	0.927*	1

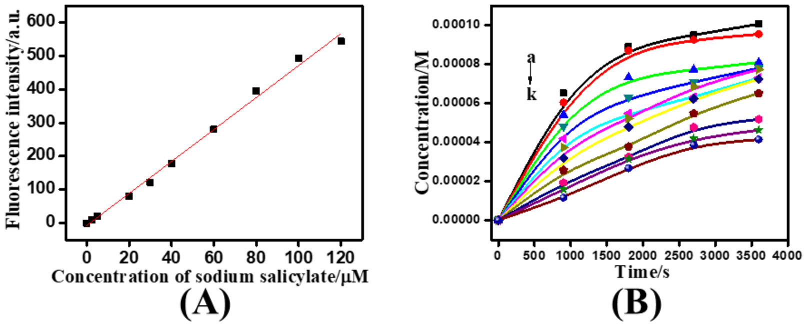

Figure S3. (A) The linear relationship between the concentration of sodium salicylate and its fluorescence intensity, (B) The oxidation kinetics of sodium benzoate with •OH.
